# Supplementary material for: One in seven pathogenic variants can be challenging to detect by NGS: an analysis of 450,000 patients with implications for clinical sensitivity and genetic test implementation
Source: Genet Med. 2021 May 18;23(9):1673–80. doi: 10.1038/s41436-021-01187-w (PMC8460443; doi:10.1038/s41436-021-01187-w)
Supplement: Supplementary file 1 — Supplementary Tables [file 41436_2021_1187_MOESM1_ESM.pdf]

Supplement to: One in seven pathogenic variants can be challenging to detect by NGS: An analysis of 450,000 patients with implications for clinical sensitivity and genetic test implementation

Authors: Stephen E. Lincoln, BS, Tina Hambuch, PhD, FACMG, Justin M. Zook, PhD, Sara L. Bristow, PhD, Kathryn Hatchell, PhD, Rebecca Truty, PhD, Michael Kennemer, MS, Brian H. Shirts, MD, PhD, Andrew Fellowes, PhD, Shimul Chowdhury, PhD, FACMG, Eric W. Klee, PhD, Shazia Mahamdallie, PhD, Megan H. Cleveland, PhD, Peter M. Vallone, PhD, Yan Ding, MD, Sheila Seal, PhD, Wasanthi DeSilva, PhD, Farol L. Tomson, PhD, Catherine Huang, PhD, Russell K. Garlick, PhD, Marc Salit, PhD, Nazneen Rahman, PhD, Stephen F. Kingsmore, MD, DSc, Matthew J. Ferber, PhD, Swaroop Aradhya, PhD, FACMG, Robert L. Nussbaum, MD, FACMG

Contents:

Supplemental Methods (This Document)

Supplemental Table S1. Pilot study variants and results (Excel File)

Supplemental Table S2. NGS workflows used in the pilot study (This Document)

Supplemental Table S3. Pilot study synthetic plasmid breakpoints (Excel File)

Supplemental Table S4. Gene List for Sensitivity Study (This Document)

Supplemental Table S5. Reference specimens used in the sensitivity study (Excel File)

Supplemental Table S6. Custom synthetic variants mixture (Excel File)

Supplemental Table S7. Findings by gene and type (Excel File)

Supplemental Table S8. Findings by gene, variant type, and order type (Excel File)

Supplemental Table S9. Common recurrent findings (Excel File)

Supplemental Figure S1. Comparison of patient and synthetic specimen NGS data (This Document)

Supplemental Files. Reference genome edits (gzipped tar file)

Supplemental Methods

The criteria for “technically challenging” variants (as illustrated in Figure 1) were:

Large indels

- In HGVS terminology: ins, del, dup, and inv variants 15 bp to 49 bp in size.
- For delins variants, the sum of the deletion and insertion size was used.

Small CNVs

- CNVs less than 2 exons in size, including:
  - a portion of an exon.
  - one complete exon.
  - one exon and a portion of a neighboring exon.
  - a portion of one exon and a portion of a neighboring exon.
- Note that some such variants will be called as complex rearrangements, rather than CNVs.

Complex rearrangements

- Very large indels 50 bp and larger.
  - Mobile element insertions are counted among these.
- Large inversions (e.g., *MSH2* exon 1-7 “Boland” inversion)
- Large variants that do not clearly fit into any of the above categories.

Low complexity

- Thresholds for defining low complexity variants were chosen empirically using the call accuracy data from our prior study of orthogonal confirmation[1] and other sources. Such variants included:
  - variants in homopolymer or dinucleotide sequences of 8 bp or more.
    - This included *MSH2*:c.942+3A>T.
  - variants in trinucleotide and longer repeats. The minimum length varied, depending on the degree of sequence complexity, but was at least 12-20 bp
    - e.g., CAGT[3] was not considered challenging but AAAG[3] was. The CAGT repeat has a 50:50 AT:GC ratio and much better local alignability than AAAG, which is also 75:25 AT:GC. Furthermore, an SNV or deletion of the G in one AAAG unit could result in a 7-8 bp homopolymer, flanked by AAAG(s), which would not happen with CAGT.
  - variants in the *CFTR*:c.1210-34 poly-T/poly-TG site and in the *ARX* GC-rich poly-alanine regions.

- Additionally, genomic regions annotated by the Global Alliance for Genomics and Health (GA4GH) stratification files[2] were included when the variant was in (not just near) a repeat
- *FMR1* trinucleotide expansions were counted separately owing to methodological differences.

Segmental duplications

- In this study, only variants in *SMN1/2, HBA1/2. GBA, SDHA, PRSS1, PMS2* (portions) and *NEB* (portions) were counted.
- CNVs within segmental duplications were considered challenging if less that 2 deleted or duplicated exons were outside of the segmentally duplicated region.

Putative mosaic

- Generally, an allele balance <20% was required as well as the absence of strand bias, known mismapping, and known artifacts.
- Such variants were subject to manual review in the Figure 2 workflow.

Notes:

The specific size thresholds we used to define “large” or “very large” sequence variants, or “small” CNVs, do not aim to precisely separate “challenging” from “not challenging” variants. Rather, we recognize that with increasing size (for sequence variants) or decreasing size (CNVs), NGS accuracy using conventional methods tends to become worse, and false-positive (FP) vs. false-negative (FN) trade-offs tend to become more difficult in test development and optimization.

Variants in low-complexity regions present particular challenges for FP/FN trade-offs. The specific bioinformatics methods and quality thresholds used in the prevalence study workflow (Figure 2) aimed to achieve high sensitivity in such regions at the possible expense of specificity, which this workflow addressed by the steps illustrated at the bottom of Figure 2 and detailed separately.[1] Other bioinformatics pipelines may find different subsets of low-complexity variants to be challenging from a sensitivity (FN) point of view, but also may exhibit very different FP rates. Workflows that do not use, or that aggressively minimize the use of orthogonal confirmation, may need to use NGS methods providing greatly diminished sensitivity in order to avoid FPs. Also, the specific biochemical steps used, particularly post-hybridization PCR, *in situ* amplification within the sequencing instrument, and read depth, are significant factors in determining which low-complexity variants are indeed technically challenging for any particular laboratory workflow.

Segmental duplications also can present variable degrees of challenge depending on the specific methods used. We examined the Global Alliance for Genomics and Health GenMap stratification files[2] and the Mandelker et al. "High Stringency" files,[3] but found that these included some regions that, for our prevalence study methods (Figure 2), were not particularly challenging. However we find it likely that these additional regions could be quite challenging for other NGS workflows, even using the same core platform (i.e., Illumina) depending on factors including read length, library insert size distribution, read depth, and alignment algorithm and parameters.

Supplemental Table S2. NGS workflows used in the pilot study

N	Platform	Coverage	Targeting	Bioinformatics	Ref
1A	Illumina 2x150	851x	Nimblegen with IDT fill-in	Aligner: Novoalign<br>Callers: GATK Unified Genotyper and Freebayes for SNVs/indels, Custom method for homopolymer regions (Coalgen), custom split-read and CNV methods (CNVitae). HGVS generation: UTA with manual review	4–6
1B	Illumina 2x150	618x	IDT	Updated version of 1A, using GATK Haplotype Caller (Freebayes remains, but only for low allele fraction variants) and other changes. See methods for details.	4–6
2	Illumina 2x100	856x	SureSelect	Aligner: BWA<br>Callers: GATK, VarScan, Breakdancer, Pindel, custom methods<br>HGVS generation: Annovar with manual review	7–9
3	Illumina 2x100	10, 680x	SureSelect	Qiagen CLC Genomics Workbench	10
4	Illumina 2x75	532x	SureSelect	Aligner: BWA-MEM<br>Callers: GATK Haplotype Caller<br>HGVS generation: Mutalyzer	11,12
5	Illumina 2x100	1264x	TruSight	Aligner: Stampy<br>Callers: Platypus<br>HGVS generation: CAVA	13–15
6	Illumina 2x150	42x	Whole-genome	Edico Dragen<br>DNA Nexus Parliament Suite (for structural variants)	16,17
7A	Illumina 2x150	849x	TruSight	Illumina MiSeq Reporter	18
7B	Illumina 2x150	849x	TruSight	Illumina BaseSpace BWA Enrichment App	19
8	Ion Torrent	420x	Ampliseq	Ion Reporter	20

Abbreviations: BWA, Burrows-Wheeler Aligner; CAVA, Clinical Annotation on Variants; GATK, Genome Analysis Toolkit; HGVS, Human Genome Variation Society; IDT, Integrated DNA Technologies; UTA, universal transcript archive.

Supplemental Table S4. Gene List for Sensitivity Study

APC	*HOXB13*	*PTEN*
ATM	*KIT*	*RAD50*
AXIN2	*MEN1*	*RAD51C*
BARD1	*MLH1*	*RAD51D*
BMPR1A	*MSH2*	*SDHA*
BRCA1	*MSH3*	*SDHB*
BRCA2	*MSH6*	*SDHC*
BRIP1	*MUTYH*	*SDHD*
CDH1	*NBN*	*SMAD4*
CDK4	*NF1*	*SMARCA4*
CDKN2A (p16INK4a, p14ARF)	*NTHL1*	*STK11*
CHEK2	*PALB2*	*TP53*
CTNNA1	*PDGFRA*	*TSC1*
DICER1	*PMS2*	*TSC2*
EPCAM (CNV only)	*POLD1*	*VHL*
GREM1 (promoter CNV only)	*POLE*	

Abbreviations: CNV, copy number variant.

Supplemental Figure S1. Comparison of patient and synthetic specimens.

(a) Alu insertion (top, synthetic; bottom, patient)

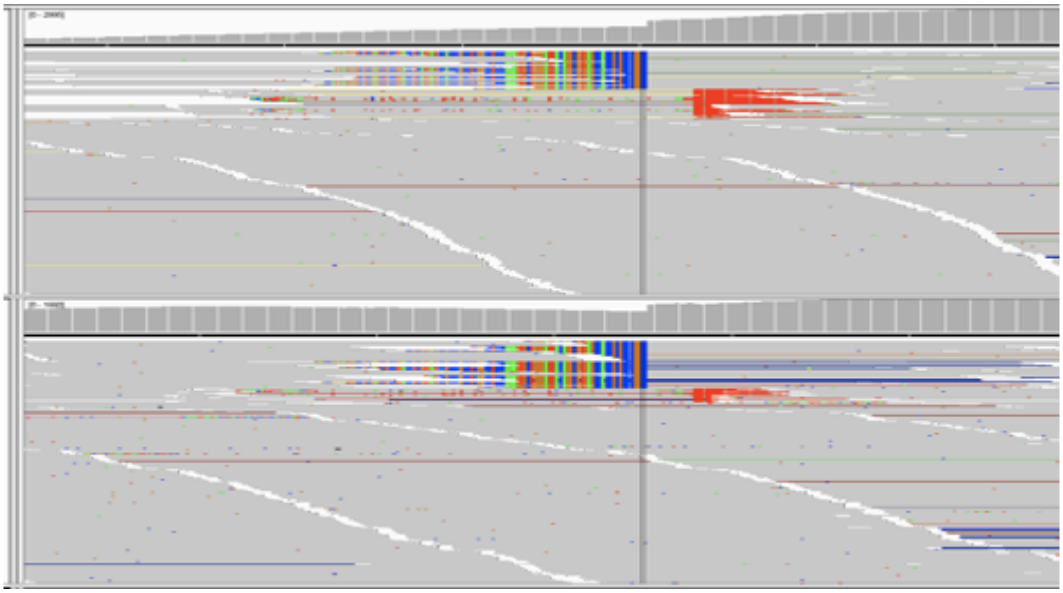

(b) CDKN2A:c.9_32dup (top, synthetic; bottom, patient)

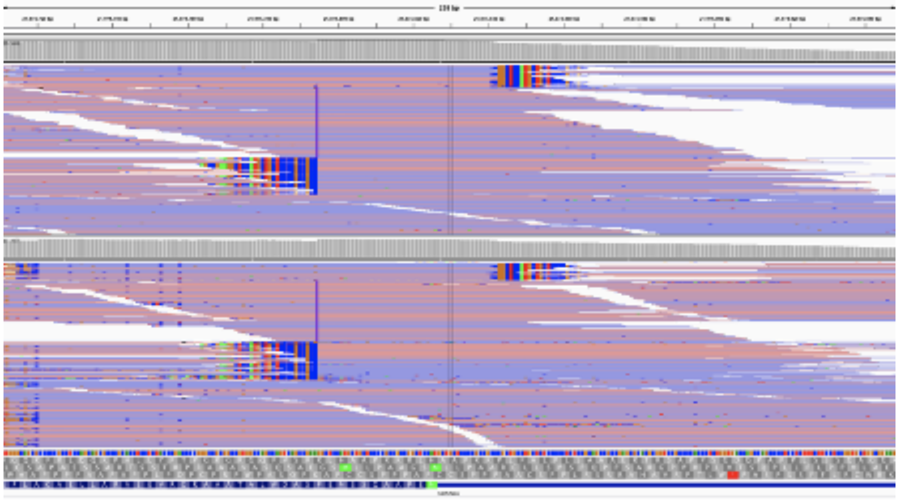

(c) MSH2:c.942+3A>T (top, synthetic; bottom, patient)

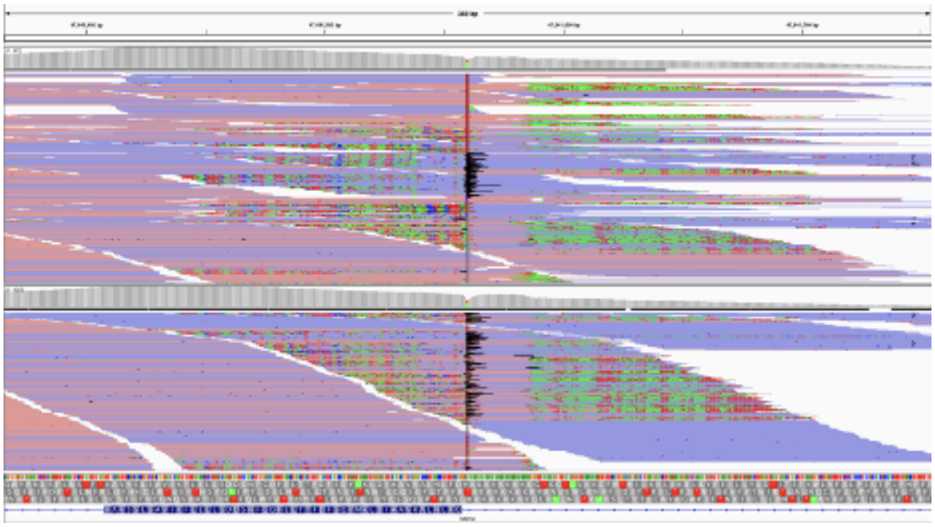

(d) Variant allele frequency of synthetic indels.

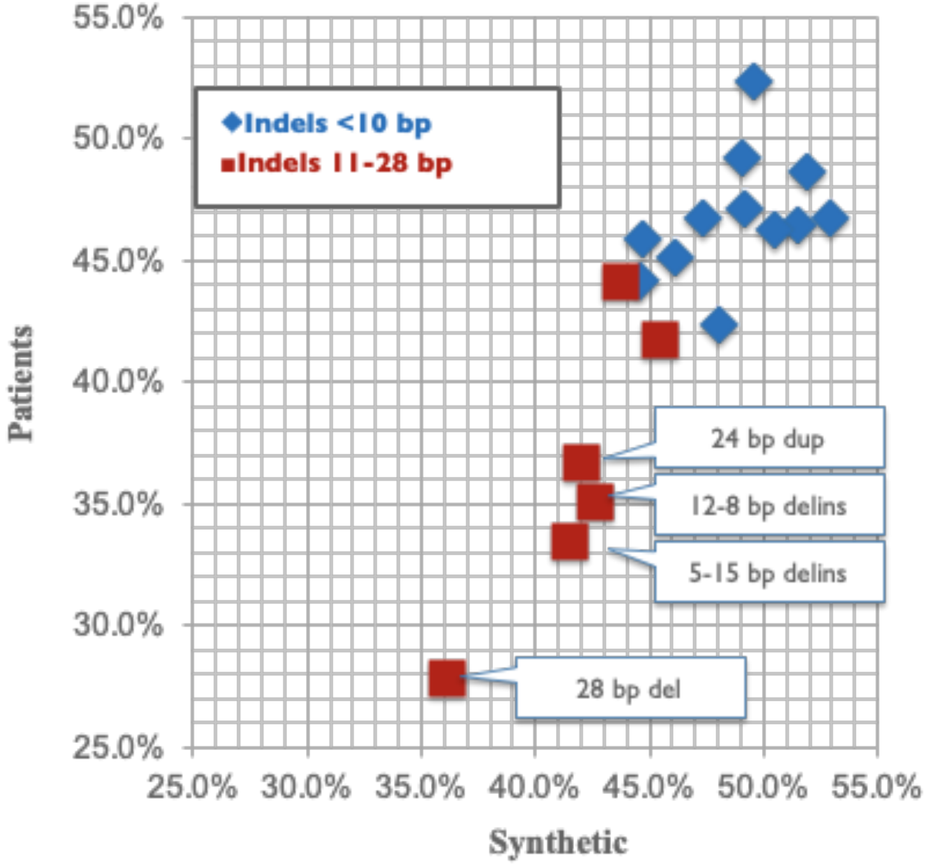

Legend: (a-c) Comparison of specific variants in the synthetic controls with the same variant in patient DNA. Synthetic variants presented similar challenges and artifacts as did their endogenous versions. (d) Plot of variant allele frequencies for synthetic indels (x-axis) with the sample variant in patient DNA (y-axis). Deviation from 50-50 was similar in the two sample types, although was slightly greater in patient specimens compared to synthetics.

Abbreviations: bp, basepair; del, deletion; dup, duplication; Indel, insertion or deletion. Delins, deletion/insertion event (also called block substitution).

References

1. Lincoln SE, Truty R, Lin C-F, et al. A Rigorous Interlaboratory Examination of the Need to Confirm Next-Generation Sequencing-Detected Variants with an Orthogonal Method in Clinical Genetic Testing. *J Mol Diagn*. 2019;21(2):318-329.

2. Krusche P, Trigg L, Boutros PC, et al. Best practices for benchmarking germline small-variant calls in human genomes. *Nat Biotechnol*. 2019;37(5):555-560.

3. Mandelker D, Schmidt RJ, Ankala A, et al. Navigating highly homologous genes in a molecular diagnostic setting: a resource for clinical next-generation sequencing. *Genet Med*. 2016;18(12):1282-1289.

4. Lincoln SE, Kobayashi Y, Anderson MJ, et al. A systematic comparison of traditional and multigene panel testing for hereditary breast and ovarian cancer genes in more than 1000 patients. *J Mol Diagn*. 2015;17(5):533-544.

5. Hart RK, Rico R, Hare E, Garcia J, Westbrook J, Fusaro VA. A Python package for parsing, validating, mapping and formatting sequence variants using HGVS nomenclature. *Bioinformatics*. 2015;31(2):268-270.

6. Invitae | Methodology & validation studies. https://www.invitae.com/en/validation-studies/. Accessed April 30, 2020.

7. Pritchard CC, Smith C, Salipante SJ, et al. ColoSeq provides comprehensive lynch and polyposis syndrome mutational analysis using massively parallel sequencing. *J Mol Diagn*. 2012;14(4):357-366.

8. Pritchard CC, Salipante SJ, Koehler K, et al. Validation and implementation of targeted capture and sequencing for the detection of actionable mutation, copy number variation, and gene rearrangement in clinical cancer specimens. *J Mol Diagn*. 2014;16(1):56-67.

9. Nord AS, Lee M, King M-C, Walsh T. Accurate and exact CNV identification from targeted high-throughput sequence data. *BMC Genomics*. 2011;12:184.

10. Biomedical Genomics Workbench direct download - Bioinformatics Software and Services | QIAGEN Digital Insights. Bioinformatics Software and Services | QIAGEN Digital Insights. https://digitalinsights.qiagen.com/products/biomedical-genomics-workbench-direct-download/. Accessed April 30, 2020.

11. Best Practices Workflows – GATK. https://gatk.broadinstitute.org/hc/en-us/sections/360007226651. Accessed April 30, 2020.

12. Mutalyzer 2.0.32 — Welcome to the Mutalyzer website. https://mutalyzer.nl/. Accessed April 30, 2020.

13. Lunter G, Goodson M. Stampy: a statistical algorithm for sensitive and fast

mapping of Illumina sequence reads. *Genome Res*. 2011;21(6):936-939.

14. Rimmer A, Phan H, Mathieson I, et al. Integrating mapping-, assembly- and haplotype-based approaches for calling variants in clinical sequencing applications. *Nat Genet*. 2014;46(8):912-918.

15. Münz M, Ruark E, Renwick A, et al. CSN and CAVA: variant annotation tools for rapid, robust next-generation sequencing analysis in the clinical setting. *Genome Med*. 2015;7:76.

16. English AC, Salerno WJ, Hampton OA, et al. Assessing structural variation in a personal genome-towards a human reference diploid genome. *BMC Genomics*. 2015;16:286.

17. Miller NA, Farrow EG, Gibson M, et al. A 26-hour system of highly sensitive whole genome sequencing for emergency management of genetic diseases. *Genome Med*. 2015;7:100.

18. MiSeq Reporter Software (MSR). https://www.illumina.com/systems/sequencing-platforms/miseq/products-services/miseq-reporter.html. Accessed April 30, 2020.

19. BWA Enrichment. https://www.illumina.com/products/by-type/informatics-products/basespace-sequence-hub/apps/bwa-enrichment.html. Accessed April 30, 2020.

20. Ion Reporter | Thermo Fisher Scientific. https://ionreporter.thermofisher.com/ir/. Accessed April 30, 2020.
